# Individualization Without Internalization

**DOI:** 10.1111/cogs.70132

**Published:** 2025-10-23

**Authors:** Ludger van Dijk

**Affiliations:** ^1^ Philosophy Group, Department of Communication, Philosophy, Technology and Education Wageningen University & Research; ^2^ Centre for Philosophical Psychology Department of Philosophy University of Antwerp

**Keywords:** Affordances, Ecological psychology, Mead, Others, Practices, Self, Skill

## Abstract

What is that “inner” voice that keeps you up at night or that tells you to stop as you reach for another chocolate? Advances in embodied cognitive science raise doubts about explaining the “self” as the result of internalizing our shared world. On that emerging view, there is nothing to transport from outside to inside the skull. But, if not an inner state of mind, then how should we understand the experience of a self? This paper develops a relational approach to individualization by aligning ecological thinking with practice theory through Meadian considerations. On this account, we continuously experience a meaningful world, filled with possibilities for action, tied to things in places and practices. Practices are intergenerational processes in which materials get organized by what we do, while in turn organizing us. Becoming a “self” requires learning to attend to such communal organizations as one's relation to the world expands across development. As we learn to engage various such organizations skillfully, we can experience them responding to us. Situated across practices, the “self” develops as a reciprocal relation between multiple timescales: notably between communal practices and a person's skilled activities. When we close our eyes and our thoughts come to the fore, we experience this reciprocal relation directly. To get this relational self into view, psychology needs to get out of our heads and study the worldly conditions that make us.

Intelligence becomes ours in the degree in which we use it and accept responsibility for consequences. It is not ours originally or by production. “It thinks” is a truer psychological statement than “I think.”

—John Dewey (1922, p. 314)

Who is that voice “in the back of your mind” telling you to stop as you are about to take a third cookie from the jar? And how come you can be surprised to find yourself eating it anyway? Where does the experience come from of having an “inner” conversation with yourself as you are contemplating, weighing the different options for your vote in the next election? How can a child at school see themselves as a failure despite their parent's encouragements? These are various experiences of a self, in the sense that one's own (future) activity is an issue for oneself. That is, one shows up as an “other” to reason about, but the person who is reasoning is that very other. Colloquially, we tend to say that such experiences of self are in our heads. But are they? And if not, how then should we think of these experiences?

Once we understand these various experiences as “inner,” it seems a matter of course that there is a process by which something external ends up being internal to our mind: a process of “internalization.” Having “internalized” our mother's disapproving voice, for example, means that it has become part of us as individuals. That we carry our mother's attitude with us when she is not there, that we see ourselves in her terms, and so on. Describing our conscience as a voice “in the back of our head” similarly evokes the idea that we, for example, internalized a public norm. Indeed, we usually do not need to talk out loud to weigh our options to vote, nor do we publicly narrate our own behavior as we take a first, then a second, and then a third double chocolate chip cookie.

Such experiences speak to an intuition of an inner self. These experiences might have a linguistic quality about them, yet they are neither spoken nor heard. Perhaps they originate somewhere behind our eyes—the position from which we have our unique perspective on the surrounding world. Indeed, we often find our thoughts coming to the fore when we close our eyes. Only when we thus learn to detach from “external contingencies,” are we able to act on our own accord (e.g., Deci, Vallerand, Pelletier, & Ryan, 1991, p. 328). But are these loosely connected experiences all somehow “inner”? Do they all point to an origin inside our heads? If so, how did the self get in there? How do we go from being an acting person in a world shared with others to having the world, others, and our own actions all contained within us?

Learning to act on one's own accord need not be the same thing as learning to act independently of “external contingencies.” Being the only one who hears a voice is not yet the same thing as it originating from “the back of our head.” Indeed, the experienced “voice” is not as clearly localizable as all that. To anticipate: why would the experience of a vague, indeterminate yet persistent voice telling you to act appropriately not be embodied by the communal activities one grows up to be a part of? Indeed, one is a part of that community whether one's eyes are open or closed.

There is surprisingly little literature in social psychology that makes the process of internalization precise (Martin, 2006; Zittoun & Gillespie, 2015; see below). More often than not, the result of development is simply assumed to be an “inner” phenomenon. The prevailing idea seems to be that there, therefore, *must* be a process by which something external gets inside our mind. Yet, as we shall see, advances in cognitive science cast serious doubt on the coherence of that view. Affirming common wisdom about the inner nature of the self thus risks empirical inadequacy: imposing undue limits on how, when, and where to study the self, we might lose sight of the very worldly conditions that make us.

This extended article aims to supply an alternative approach to the various phenomena of a self, one that does not assume that these all play out in our heads. Exploring what is meant by “internalization” in psychological literature, Section 1 argues that cognitive science conflates internalization as an individual's experience of a self with the process by which we explain such experiences. As for the latter, the emerging view coming out of enactive‐ecological theorizing about cognition is that there is nothing—literally *no thing*—to transport from outside to inside our heads (e.g., Gibson, 1979, p. 57; Hutto & Myin, 2013; Oyama, 2000). Cognitive systems do not process bits and pieces of informational content. On that view, cognitive systems cannot be thought to construct inner images of one's self and others to represent for deliberation.

The alternative requires a *relational* instead of representational starting point. Section 2 provides such a starting point by taking a page from ecological psychology. Ecological psychology considers the world in relation to an organism or human as practically meaningful: it affords sitting, eating, burning, shelter, safety, or support. The approach has been successful in perception and action research (e.g., Turvey, 1990; Warren, 2006; see Bruineberg, Withagen, & Van Dijk, 2023 for discussion). I will argue that ecological insights can be applied much more broadly if we consider the relational process in which we experience the world to be constituted over time. I will call this process a “temporally reciprocal” relation. In this relation, we experience our *current* environment in light of the *past* involvement it continues and for the *futures* it opens up.

Such a temporal relationalism suggests a methodological reorientation when we use it to approach self‐formation. Instead of going inward, supplementing current involvement with representational processes inside the head, inquiry goes wide. The more personal or individual an experience becomes, the larger the timescales of involvement that we need to keep track of to see its relational reality. Section 3 introduces practice theory as providing the concepts to bring these larger organizations into view. It shows that, as many people engage their surroundings across generations, this gives rise to larger‐scale organizations that we call “practices.” Such practices, I argue, organize the meaningful world into affective and normative places and settings.

The article then proceeds to connect the combined ecological and practice‐theoretical perspective with George Herbert Mead's theory of self‐formation. Mead's work is commonly acknowledged to explain that the source of what ends up in our heads is social, public, and shared before it becomes internal, personal and private. The claim that this paper develops goes one step further by denying that the self ever ends up in the head: we instead become individual persons in the shared world. While not the only precognitivist thinker that allows us to rethink the notion of internalization (e.g., Vygotsky, 1962; 1978; see below), Mead's work is of special importance to this paper because of the pragmatism that animates it, and which it shares with ecological psychology. As we shall see, Mead developed a dedicated vocabulary that is particularly well‐suited to understand in temporal terms, and thus helps to align practice theory and ecological thinking.

Section 4 considers what happens to a person's experience if the temporal reciprocal relations with their surroundings include their own voice responding to others and to their own actions: the temporally extensive process thus becomes “self‐selective.” With Mead's help, Section 5 supersizes this reciprocal process by considering how it gets reorganized in a shared world of practices. Expanding their involvement, a person learns what places, settings, and practices require of them. Learning to notice how these organizations respond to an individual's contributions, these organizations, in Mead's words, respond as “generalized others” to some*one*, whose future activity is increasingly anticipated.

Section 6 brings all these threads together by considering Mead's famous distinction between the “I” and the “me” in ecological terms. It suggests that the acting *I* and the reflecting *me* emerge together in development as the temporally reciprocal relation introduced in Section 2 meets the shared world of practices considered in Section 3. On that reading, the self cannot be understood outside the shared world of practices that it forms out of and to which it contributes. We do not “internalize” the world inside us, but the reorganized world *individualizes* us as we differentiate ourselves in it. Finally, Section 7 concludes by returning to the methodological reorientation that this view of individualization without internalization brings: instead of assuming that experience plays out in the head, and deploying methods to match, a relational approach will urge us to pair close observations of an individual's engagement over time with an eye for the wider world that continues to make us.

## 1 Internalization as process and outcome

Aiming to develop an alternative to “internalization” is a positive project. Yet, there are powerful arguments against the coherence and explanatory purchase of a process of internalization too (Costall, 2011; Hutto & Myin, 2013; Keijzer, 2001; Ramsey, 2017; Van Orden, Pennington, & Stone, 2001). Let me briefly rehearse one of them in this section to motivate why cognitive science might want to entertain alternative conceptions.

Internalism about the mind reverberates throughout psychology. It informs our conception of even the most basic perceptual processes. Cognitive psychology, for example, considers perception as an “internalization” of the external world: we are stimulated by a bright light, that stimulation hits the eyes and travels along the optic nerve to be “processed” by our brain. What gets inside is “information” about the world from which we reconstruct the cause of the stimulation, say, a candle. Much of cognitive science starts from the premise that once we have information inside our heads, our brain or mind creates a model, a schema, or a representation of the world, which we then use to reason about said world (Helmholtz, 1878; see Courville, Daw, & Touretzky, 2006; Ma, 2012).

Assuming that the mind is an inner affair, it seems humans must have evolved mechanisms in our heads that not only represent one's personal surroundings but also include humans’ “collective intentionality” (Tomasello & Rakoczy, 2003, p. 139; Tomasello, 2016; see Nungesser, 2020, for a nuanced reading). There are, for instance, influential accounts that claim that by learning the shared use of symbols, a child “cognitively represents not just the perceptual or motoric aspects of a situation, but also … that the current situation may be attentionally construed by ‘us’” (Tomasello, 2003, p. 53). That is, we internally represent the responses of people external to us: our mind takes in and organizes the responses of others that we encounter, combines them, and commits the result to memory as a generalized whole (Valsiner & Van der Veer, 1988).

The main problem for such theories of internalization that I see is that they rely on a notion of “information” or “representation” that reifies an (possible) outcome of a developmental process to its prior existing source. Reification is a common phenomenon in (psychological) science and has been criticized numerous times (Dewey, 1896, p. 367, 1910, see Section 2.1; James, 1890, p. 196, 1907, p. 115; see also Harris, 1981; Heft, 2003; Ingold, 1993; Noble, 1981; Oyama, 2000; Shotter, 1983; Van Dijk, 2016; Van Geert & de Ruiter, 2022; Whitehead, 1925). A problem with reification is first that the entity, once reified, is placed outside of the process in which it is found. It lacks a developmental history and becomes a dogmatic commitment rather than a tractable hypothesis. Second, a theory that relies on reification risks becoming empirically vacuous, because its explanation becomes circular. Something cannot do interesting work to explain a phenomenon and be the phenomenon in need of explanation at the same time (see Van Orden et al., 2001).

Think, for example, of Molière's famous 1673 play in which he made fun of the medical profession of his time. Physicians used fancy Latin words to cover up their lack of knowledge. Asked why opium puts people to sleep, they explain that opium has a *virtus dormitiva*, a special sleep‐inducing power. Of course, saying that opium puts people to sleep because it has sleep‐inducing powers is no explanation at all. Molière was thus pointing out the circularity of explaining the eventual outcome of opioid use by assuming a prior state that already contains the outcome in advance of it.

Informational “content” is psychology's *virtus dormitiva*. Information is said to carry content if it provides a description, picture, or model that can be true or false (correct or incorrect, etc.) of the thing described, depicted, or modeled. In the theories above, such content traveled inside through the senses, was combined with other pieces of information, integrated into representations, stored in memory, and so on. Yet, as philosophers Dan Hutto and Erik Myin forcefully argued: there is to date no naturalistic (i.e., nondualistic) and noncircular account of these original, nonderived states of content (Hutto & Myin, 2013). What they call the “hard problem of content” is that such an account of content is, in fact, impossible. If this is right, then psychology simply cannot help itself to the informational building blocks necessary to get the process of internalization off the ground.

Without informational content that travels inside the mind, without bits of information to process, integrate, manipulate, or store, what is left of “internalization” to account for becoming a self? To emphasize, this question is not to deny the reality of the various experiences that we call “inner.” Rather, taking the various experiences of “another to one's self” seriously, the question is how we understand the development of an individual's various particular and personal experiences without attributing them to an inner realm.

Whatever we think about the argument against representational content, it seems prudent to have an alternative. With the developments in embodied‐embedded cognitive science, such an alternative is in the cards (Chemero, 2009; De Haan, 2020; Di Paolo, Buhrmann, & Barandiaran, 2017, 2018; Gallagher, 2017, 2021; Malafouris, 2013; Withagen, 2022). On the view developed here, becoming an individual, experiencing one's self, requires *more*, not *less*, involvement with the world. This paper aims to supply a way of thinking about becoming an individual that keeps the ongoing relationships of involvement in view. Instead of talking about “internalization,” I will, therefore, suggest to talk of “individualization.” To repeat, the aim is not to explain (away) the experiences that cognitive science traditionally assumes to be internal. Rather, it is to thematize the world‐involving relations that these experiences depend on, so that cognitive science might look beyond the brain, and seek noncircular explanations through (qualitative) methods that bring worldly involvement across timescales into view.

Combining ecological psychology (Section 2) and practice theory (Section 3) with Mead's theory of self‐formation (Sections 4 and 5), this paper claims that as we develop a self, we do not internalize the world into our private realms, but instead the shared world is individualized by our activities (Section 6). I will return to the methodological implications of the relational view this paper proposes in the Concluding Remarks (Section 7). The first step to this view of individualization without internalization is, however, to move away from representationalism and adopt a relational understanding of experience.

## 2 A relational world

Instead of assuming that there is an external world that somehow needs to be represented internally, ecological psychology starts from the assertion that the animal and the environment are reciprocally dependent on one another: they make an “inseparable pair” (J. Gibson, 1979, p. 8; Costall, 2004; Heft, 2007; Turvey & Shaw, 1999; Warren, 2006). It is in the coordinative activities that maintain their mutual relation that the animal and the world continuously take shape. Starting from this mutualism, two more features follow.^1^ First, within this mutuality, the world that is taking shape is considered in pragmatic terms. James Gibson coined the term “affordance” to emphasize that the world is experienced for the practical differences it can make throughout an animal's life (J. Gibson, 1966, p. 285, 1979, p. 127). Affordances are possibilities for action offered by the environment. For a human of a fitting size and shape, a chair invites sitting when tired. For an albatross, a cluster of fish eggs affords eating. Ants can walk a razor's edge that would cut our fingers. Understanding the environment in relation to an organism allows us to think that meaning and value are not (somehow) mentally constructed, but are an intricate part of the organism's relation to their surroundings.

Affordances shift psychology's attention from the isolated organism (or its brain) to the wider world in which the organism lives. When a child burns herself on a candle flame, for example, she is not learning to project or add value to a meaningless stimulus. She instead learns how a flame practically relates to her: it means pain when contact occurs. Starting from the relation organisms develop with their surroundings, they experience the world in its immediacy. Affordances aim to capture this relation.

Second, then, the ecological approach suggests that perception is not representational but rather *relational* in nature (Chemero, 2009; Costall, 1995, 2004; Dent‐Read & Zukow‐Goldring, 1997; J. Gibson, 1979; Heft, 1989, 2001; Heras‐Escribano, 2019; Rietveld & Kiverstein, 2014; Stoffregen, 2003; Szokolszky & Read, 2018; see Bruineberg et al., 2023 for other views). The world is a constituent of experience—not a cause of it (J. Gibson, 1979, p. 239; see Heft, 1989, 2001; Noble, 1981; Shotter, 1983; Van Dijk & Rietveld, 2018). It is this relational conception of mind that undercuts the need for mental mechanisms that internalize and manipulate content about the world. If the world is already an intricate part of the relation that we experience, then there is no need to double the world elsewhere in the system. Ecological psychology thus takes perception to be *direct* (e.g., Chemero, 2009; Michaels & Carello, 1981; Turvey, 1992).

### 2.1 Expanding relations in time

How does this relation with the environment become available to the organism? To see this, let us turn to John Dewey's work. Dewey's analysis of the active process of perceiving is important both for the criticism of the tradition it contains as well as for the positive alternative it opens up. Dewey, moreover, has had a lasting influence on the ecological approach (J. Gibson, 1966; see also Costall, 1995; Heft, 1989; Ingold, 2018; Noble, 1981; Shaw & Turvey, 1981). Dewey considered the above case of the child burning herself on a flame in some detail. Traditionally, this event was thought to partition into first, a flame being a stimulus for the child. This stimulus was followed, second, by a reach in response to it. The reach causes a painful burn, which is, third, another stimulus that, forth and finally, causes a withdrawing response. The picture that emerges is of an iterative process of stimuli traveling inside and up, from world to mind, and responses traveling down and out, from mind to world.

To Dewey (1896), such a story of stimuli ignores a crucial question: Why is the flame a “stimulus” in the first place? Notice that this question exposes the stimulus‐response theory as a *reification* of the very process it means to account for: the developmental process by which the flame eventually comes to function as a “stimulus” (Dewey, 1896, p. 367). Talk of stimuli and responses takes for granted the very developmental coordination needed for them to be understood as such. That is, like the conception of information discussed in Section 1, stimulus‐response theory reifies an (possible) outcome of engagement to its prior existing source. Dewey, however, also offers a positive alternative account.

On Dewey's view, the enticing nature of the flickering light needs to be understood amid a wider, ongoing coordinated activity: “The real beginning,” he wrote, “is with the act of seeing; it is looking and not the sensation of light” (Dewey, 1896, pp. 358–359). Burning a finger is not connected by an internal mental process to the sensation of light, but it is an ongoing development of the flame entering into activity by looking. Over the course of the child's activity, undergoing the painful burn develops the act of seeing a light into the “seeing‐of‐a‐light‐that‐means‐pain‐when‐contact‐occurs” (Dewey, 1896, p. 360).

In Dewey's analysis, we find a reciprocity that is not limited to the relation between the worldly stimulus and the child's response. A *second* reciprocity emerges across two timescales. What we call “stimulus” and “response” are small yet extensive processes unfolding within a larger‐scale coordinative activity. Neither the two smaller‐scale processes nor this larger‐scale coordinative activity is, however, fully determinate at a single point in time. They require each other and thus codetermine over time. By responding, the child's activity is not only determining the “stimulus” but also concurrently determining the much larger scale of activity out of which both the stimulus and its response emerge (Shotter, 1983). It is for this reason that, for Dewey, withdrawing the hand was a response “not merely *to* the stimulus; it is *into* it” (Dewey, 1896, p. 359).

**Fig. 1:**
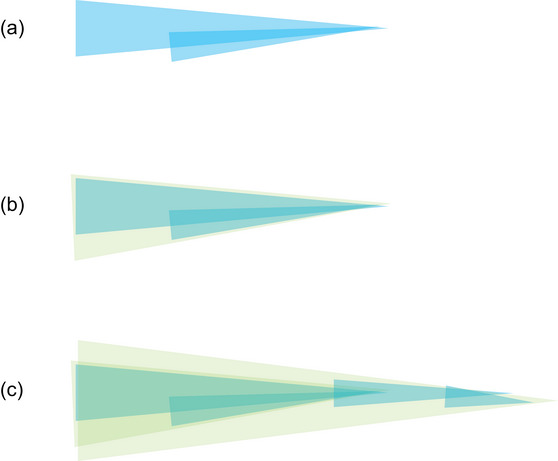
The world‐involving coordination getting ahead of itself. These figures are based on Van Dijk and Rietveld (2018, fig. 2) and Ingold (2011, p. 69). (a) The “act of seeing,” represented by the upper light blue line is continued by the “act of reaching,” the lower smaller line joining. Each line points to the right to suggest a directional tendency to the process unfolding. (b) Meanwhile, as the two activities coordinate, they form a larger‐scale activity: “seeing‐for‐reaching,” here drawn as a wider but also green line superimposed over the blue lines: the small‐scale activities lay down a path in developmental time. (c) This larger‐scale activity with its directional tendency conditions new activity: seeing‐for‐reaching sets up the conditions for experiencing a painful burn and the hand withdrawing (the two additional small blue lines). This transforms the larger timescale of seeing‐for‐reaching into “seeing‐of‐a‐light‐that‐means‐pain‐when‐contact‐occurs” (Dewey, 1896, p. 360). This larger scale of activity is again drawn as a thinker green line superimposed over the smaller lines; it is neither contained in the head nor on the flame, but is this temporally extensive relation. Notice that the coordination depicted is a world‐determining activity. While going from a to c, the figures indicate an open‐ended process in which an indeterminate yet enticing light makes way for a flame that affords pain to the child. Afterward, however, we can also look back on the process, reading figure c now from the right to left, for example, and see only the determined materials and the actions that have unfolded. Dewey suggests that when we, as psychologists, analyze the ongoing process in terms of “stimuli” and “responses,” we conflate our own familiarity with this finished process with the child's actual involvement still ongoing.

We can think of the developmental process Dewey describes as a *temporally reciprocal* process (Van Dijk, 2020; 2021). A temporal reciprocity is a relational process in which at least two relata concurrently take shape over different timescales. Dewey describes a temporally reciprocal process because (a) the coordinative activity enables the light to matter, and the burn to hurt, while (b) the light and the burn achieve continuation of the child's coordinative activity with the world in a particular way. As the child is invited by the flame (“stimulus”) to reach for it (“response”), the flame enters into the activity of the child (“coordination”). In this case, it hurts her. Together, this shapes the extensive coordinative relation to flames further: while enticing the flame also becomes fearful and, as the child becomes more skilled, she trusts the next encounter with flames to hurt if not careful. Across development, the child is starting to partake in a *larger‐scale* worldly relationship. See Fig. 1 for a visualization of this multiscale process.^2^

Dewey's developmental approach turns psychology inside out: the child's experience is not of a world represented in her head, but she learns to experience her being‐in‐the‐world. She experiences her relationship with the temporalized world of affordances *directly* by participating in it. Thus, the idea that “stimuli” need to get into the head evaporates (which is not to say that the physiological processes in the head and body are not crucial to maintaining that relationship, see the final section). On this view, to experience more of the world, the child would need to let her temporal relation with the world expand and refine.

Dewey's analysis shows how the affording world is discovered in ongoing coordinative participation. Over time, the child's world‐involving activities tie in with the meaningful and affective world in more and more refined ways. Across development, the coarse experience of an enticing light is refined to an experience of dangerous flame affording a painful burn (Heft, 1989). The child, in short, learns to join in, and continue, the affording world. As she does, the unfolding relation with her surroundings grows and sets up new futures to explore. A temporally reciprocal relation, then, continuously “gets ahead of itself” expanding into new material (Van Dijk, 2021).

This brings us back to Gibson's perceptual psychology. Gibson too criticized the idea that there is anything to isolate as stimulus or response in the flow of everyday life (J. Gibson, 1960). To Gibson, too, perceiving is an ongoing developmental process. The perceptual relation grows “wider and finer and longer and richer and fuller” as we learn to engage our environment (J. Gibson, 1979, p. 255). We are able to engage the environment in more and more elaborate ways over time because we maintain expansive relationships with and in our environment across development. The larger and wider our worldly relationship grows, the more refined experiences open up to us (Withagen, 2022). One of those experiences, as we shall see below, is that of “becoming another to one's self.”

Crucial to a relational account of self will be to recognize that the temporally reciprocal relation described includes an indeterminate yet very real future (Van Dijk, 2021). That open‐ended future of the organism−world relation is the directional tendency that the reciprocal process gains over time. As part of the relation, we experience this directional tendency as, say, a flame so enticing that it draws a child to reach for the light or as a chair inviting to sit when tired. These are experiences of an open‐ended, ongoing relation with the world. They need neither be attributed to the world (the flame, the chair) nor to the organism alone. Experiencing stops nothing short of the (unfinished) relation as a whole. The affording world is a constitutive part of the perceptual process, not merely a causal factor.

Indeed, it is not just an open‐ended future that is thus directly experienced. Inside the developing reciprocity, the *current* environment that we engage is indeed experienced for the potential *future* activities it invites. But this relies on a *history* of previously established relations that one is continuing in their worldly coordination. This history of engagement continues to be a part of the larger timescale that makes up the temporally reciprocal relation. Within a temporally reciprocal relation: “the feeling of past, present and future are merged or, more exactly, the activity of perception is acknowledged to be retrospective and prospective” (J. Gibson, 1975, p. 300; for a detailed analysis, see Van Dijk, 2021; Van Dijk & Rietveld, 2018; Van Dijk & Withagen, 2016).

### 2.2 Sharing the world

**Fig. 2:**
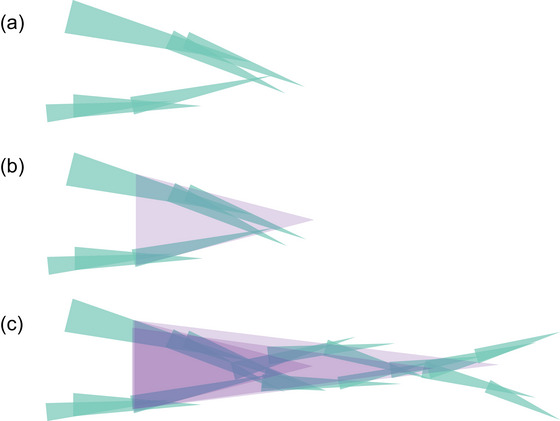
Two materially engaged people joining in a conversation. This figure draws on Ingold's drawing (2013, p. 107), but aims to foreground how coordinating together can give shape to larger and shared timescales of involvement. Notice that this representation builds on Fig. 1, in which the greenish color was the larger‐scale developmental process shaping over time as worldly coordination took shape (in blue). For ease of presentation, these blue lines are suppressed here. (a) The two situated persons with their own history of involvement, represented by two greenish lines, coordinate their activity. (b) Meanwhile, as the two people coordinate their doings and sayings, they form a larger‐scale activity: a conversation. The conversation is drawn as a wider purple line superimposed over the green lines to indicate that the two people's activities shape each other by shaping the joined conversation. (c) This conversation continues with the activities that its directional tendency sets up. This larger scale of activity is again drawn as a thicker purple line superimposed over the smaller lines. The larger‐scale conversation that is participated in is hence open to experience (see main text for details). Notice that, as the conversation draws to a close, both situated people come out redirected, if ever so slightly. Conversations are typically situated within even larger timescales still. These are considered below (cf. Fig. 3).

Temporally reciprocal relations are not restricted to individual development. We find them everywhere. Indeed, as we shall see, they exist across multiple timescales and are often maintained by multiple people across space and time. An everyday example is having a conversation (Di Paolo, Cuffari, & De Jaegher, 2018; Goodwin, 2018; Mead, 1934; Richardson, Dale, & Kirkham, 2007; Sacks, Schegloff, & Jefferson, 1974). Everybody who has felt a conversation slip into a fight (but powerless to stop it), or sensed a discussion drawing to a close (and so best not bring up a new topic), recognizes a directional tendency to the conversation beyond the utterances of each individual involved. Such a conversation, too, is a temporally reciprocal organization. That is, (a) the conversation developed so far sets up the conditions for some, and not other, small‐scale utterances to be appropriate. In turn (b), these utterances provide continuation to the larger unfolding conversation. Notice that, just as the flame could not be understood as a “stimulus” in isolation from the child's development, in the case of a conversation, any doing or saying is likewise indeterminate at a single point in time when isolated from the wider process it helps to shape for everyone involved. See Fig. 2.

What a facial expression, an utterance, or a gesture means inside such shared situations is not given, but gets determined inside this multiscale process. Hence, in what Mead called “co‐operative activity,” the (linguistic) activities are constitutive of the larger scale of coordination so that in conversation:

The beginning of the act of one is a stimulus to the other to respond in a certain way, while the beginning of this response becomes again a stimulus to the first to adjust his action to the oncoming response (Mead, 1934, pp. 144–145).

If we take the temporal organization of a conversation seriously, Mead's insight can be understood to suggest that it is through the larger‐scale process, which mutually constitutes both participants’ utterances and gestures, that they anticipate each other's activities by adding theirs. Inside the larger‐scale process that is the conversation, one's active involvement is thus co‐constituting the future response of the other. Participating in a conversation, people can thus experience this determining relationship directly:

We are finding out what we are going to say, what we are going to do, by saying and doing, and in the process we are continually controlling the process itself. In the conversation of gestures what we say calls out a certain response in another and that in turn changes our own action, so that we shift from what we started to do because of the reply the other makes (Mead, 1934, pp. 140–141).

Our doings and sayings “call out,” or invite, a certain response of the other because of the larger conversation that shapes both participants—in Fig. 2, this larger organization is depicted in purple. By co‐operating, participants redirect each other toward the tendency of this larger whole: orienting them both along the conversation's shared directional tendency (hence the invited response is better thought of as “of” than “in” the other). Thus, we feel a conversation slip into a fight or know exactly what to say or anticipate how the other will respond.

It is the acknowledgment of the reality of these larger organizations, to which a person contributes their activity, that makes “inner” mental states to understand the emergence of the self vis‐à‐vis another superfluous. Instead, we share the world in increasingly large temporal organizations with each other. These organizations in which we participate, in turn, shape what we do and notice. Over time, as we shall see, we can learn to differentiate our own unique contributions to this reorganized world of “practices.”

## 3 Practices

It is not just the child who has a history that sets her up to be invited by a flame. Candle flames are part of a practice that humans cultivated for millennia. The candle is a sociohistorical achievement: developing out of oil lamps known to be used for over 8000 years. A modern candle, made from paraffin, a byproduct from the mid‐19th century oil industry, allows for a highly controlled and relatively clean burn. While candles are no longer needed for heat or light in the Western form of life, they do afford creating a beautifully lit atmosphere, used for fancy dinners, romantic evenings, religious ceremonies, and so on. The child does not encounter the flame in isolation from such circumstances. She might, for instance, be seated at a table, during a fancy dinner with her parents and their friends. That dinner setting is structured by materials, chairs, a table and cutlery, all organized according to the norms of our dining practices. Likewise, the dining room is separated from, but dependent on, say, a kitchen. In short, from candles to kitchens and from paraffin to dinner parties, human activity coordinates things into organizations distributed across time and space.

These organizations are called practices. Practices are intergenerational processes in which materials get organized by what we do. But, as we shall see, practices concurrently also organize us (Costall, 1997; Costall & Dreier, 2006; Reckwitz, 2012; Schatzki, 2002). Examples include “farming practices, business practices, voting practices, teaching practices, celebration practices, cooking practices, recreational practices, industrial practices, religious practices, banking practices” (Schatzki, 1996, p. 98). Our human lives engage many practices, often at the same time.

How do these organizations take shape? According to practice theory, practices are material organizations constituted in the skilled activity of many people over time (Schatzki, 1996, p. 91). One particularly important skill used to coordinate materials across space and time is our human languaging skill. The view of languaging adopted here has nothing to do with the idea that we have intrinsic content in our heads, which we put into words in hopes of others deciphering and building up in their heads the same mental contents. With ecological psychology (e.g., Cowley, 1994, 2011; Fowler, 1986; Hodges, 2007, 2014; Rączaszek‐Leonardi, 2016; Van den Herik, 2018), but also distributed and interactive approaches (Harvey, 2015; Steffensen, 2013), semiotics (Goodwin, 2018), integrational linguistics (Love, 2017; Taylor, 2000), and enactivism (Cuffari, Paolo, & Jaeger, 2015; Di Paolo et al., 2018), we can consider language use primarily as directing and shaping the flow of situated activity.

Languaging skills afford us to quickly set up conditions for future activity beyond the current setting. Making promises, planning dinner, writing an email, praying for salvation, or calling attention to a bird on the lawn, these are specific languaging activities that rely on a larger‐scale recurrence of activities and situations in order to make sense. In turn, such languaging thus organizes the world: it sets up shared ways of doing things and hence “bundle” situated activities into material practices (Schatzki, 2002, p. 71).

We can understand this bundling in temporally reciprocal terms. Specifically, (a) a practice enables certain doings and sayings by skilled individuals across specific situations, while (b) skillfully acting across those situations achieves continuation of the practice in a particular way. For instance, by making promises and keeping them (“let me make you dinner next week”), or giving orders and following them (“one ounce of Picodon de la Drôme please”), we rely on and maintain the recurrence of dinners and cheese stores with their specific forms of involvement. At the same time, these situations coordinate into, say, celebration practices or cooking practices.

The practices that thus emerge have again a temporal existence beyond any single person's activity or utterance contributing to them. Practices instead are shared ways of doing things: there is a general way to act in a grocery store or to make lasagna. That is, “how one does things.” I want to stress, however, that practices are always world‐involving: they are tied to places and things. They are *ways of doing things with things*, to build on Alan Costall's and Ole Dreier's expression (Costall & Dreier, 2006). Indeed, in the temporal relation explicated above, the things we encounter and how we engage with those things is reciprocally dependent:

Things are best understood … not as fixed and independent of people, but as themselves transformed, even coming into being, within ongoing practices, and which these objects, in turn, transform. We … learn more about both people and things by studying them as worldly, not just as in the world, but as incorporated into practices in the world (Costall & Dreier, 2006; see Costall, 2012).

**Fig. 3:**
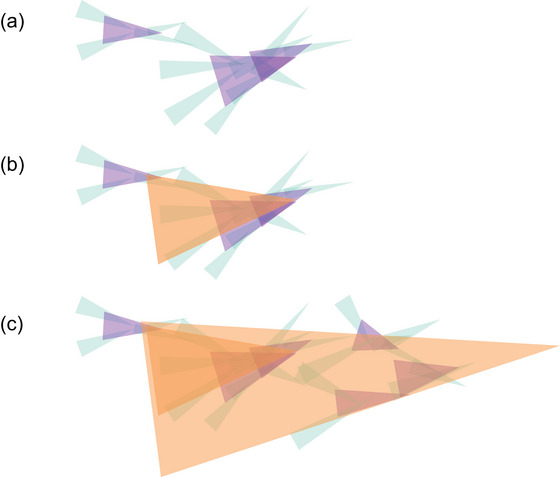
Bundling activities into practices over time. (a) On the left, two people situated in ongoing activity (in green) shaping a conversation (purple), transitioning, on the right, to four people in a shared situation (such as a behavior setting, see Section 3.1). In both cases, the people direct and are redirected by the larger timescale that constituted their shared situation. (b) Meanwhile, as these conversations and settings unfold they bundle together to form a yet larger scale of coordination: a practice. That emerging practice is here drawn in orange. This depiction is rather schematic. Practices unfold over much larger timescales than this depiction suggests: they can be stabilized across years of recurring settings, across generations of participants. Moreover, any situation will likely require and feed into multiple practices (see main text). I have suppressed these complications. (c) The practice, with its directional tendency, grows over time to give shape to the shared situations and conversations that shape their participants. In Section 4, we will begin to zoom in on a particular person within this multiscale organization and consider how this organization organizes them.

In short, practices are communal patterns of material involvement, achieved and maintained by the skillful doings and sayings of countless skilled participants across multiple generations. To act skillfully depends not just on a sensitivity to the needs of the current situation, but also on a sensitivity to the overall tendencies of the larger organization that the situation adds to. In between, we find conversations in places and shared settings that humans spend so much of their lives in. The relations among these concepts are visualized in Fig. 3.

### 3.1 Doing things in places

A person's concrete doing, saying, and noticing is situated within communal practices that they, in turn, help to continue. These reciprocal processes take materials along for the ride: organizing candles and dinner tables, for example. Or hammers, wall and nails, binoculars, and bird guides. Things get organized to be used in particular ways, and at particular places and times. A chair affords sitting when tired. But in a museum with a Rietveld chair on a pedestal, that chair does not (Withagen, Poel, Araújo, & Pepping, 2012). A museum, in fact, is a place of action, differentiated from other places for what we can do and humans learn to experience them as such (Lucas, 2024).

Andreas Reckwitz calls this reorganization of our (affording) world into places a “spatializing”: a making of spaces (Reckwitz, 2012, p. 252). Such spaces are not mere physical locales, but temporal organizations that, like the affordances they organize, are meaningful or *affective*:

Every complex of social practices … implies a form of affective space. In modern societies, this spatialising often results in built, architectural spaces that are made for and correspond with specific practices. Economic, political, private and educational practices are connected with particular built spaces, which the practices handle and which in turn influence these very same practices (Reckwitz, 2012, p. 254).

There is then a reciprocal dependency between organized material stabilities, in space and time, and the practices in which they form. On a practice‐theoretical approach, affectivity belongs to this temporal relationship, not to any of its constituents in isolation. Indeed, places such as cheese stores, dinner situations, or a museum are reorganizations of our affective world—a meaningful world of affordances. Often, these organizations of materials and people, over time, do not depend on any particular individual's activity, but the individual's activities do rely on the availability or recurrence of these organizations within a practice.

Ecological psychologist Roger Barker also noted the meaningful and, indeed, normative organization of places of action, which he called “behavior settings” (Barker, 1968; see Avram, Jones, Lucas, & Barrett, 2024; Di Rienzo, Myin, & Van Dijk, 2024; Heft, 2001). Our practices set up an endless variety of behavior settings: classrooms, dinners, restaurants, carpet shops, distribution centers, soccer matches, waiting rooms, doctors’ appointments, libraries, theaters, picket lines, religious services, and so on. Behavior settings are situations with definite spatial but also temporal boundaries that are achieved and maintained by the collective activity of different people, yet require no one person in particular (Heft, 2007, p. 98). That is, such settings emerge, exist for a while, and dissipate. But crucially, they recur regularly over time, and often across different places, and hence fortify the practices in which they shape (see Fig. 3).

A behavior setting, such as a wedding, may be a once‐in‐a‐lifetime event for some of its participants, while others will add their skills to such settings every few days. Some behavior settings recur once every few years in one region but weekly across the globe (e.g., furnishing our voting practices). We hope to visit the dentist's chair as little as possible, but the dentist is a part of the setting four times an hour. Likewise, a storekeeper maintains a standing pattern of behavior with different customers for much of the day, but anyone will contribute to the grocery setting perhaps once a week. A great many settings, however, recur day after day for most of us; breakfasts, dinners, riding the train, sitting in class or at the office, having small talk at the coffee machine. They structure much of what we do and notice in everyday life.

Connecting Barker's ecological psychology with Schatzki's and Reckwitz's practice theory, suggests that by learning to engage places and settings—learning, for example, how to behave during dinner, or what to say in a cheese store—we gain a sense for the larger practices in which these settings live. Reciprocally, learning one's way about these practices, developing skills, we sense what is appropriate to do in the next setting; we learn what to notice or to ignore. Because of their reciprocal dependency, we learn to sense what is better or worse to do in a situation relative to the values of its larger practice—that is, we gain an experience of *normativity*, of “oughtness” or “acceptability” (Schatzki, 2002, p. 80; see Rietveld, 2008). We get to experience such a feel for the demands of a situation as we engage it because our activity is a constitutive part of it. That is, the contention is that practices shape us very concretely, in what we do, feel, and notice. We can experience practices directly because we participate in them.

Practices, as ways of doing things with things, include ways of doing, feeling, and noticing. They embody “attentive norms” to which participants grow sensitive and contribute (Crary, 1999, p. 5). On that view, for example, the norms for good bird watching reside neither in the heads of the person nor in the pages of a field guide. Rather, one's observations get increasingly organized by what the book points out as it successfully guides the person to an identification in the field and, conversely, one gets better at identification in the field as the subtle ways of using the guide, its significances, and so on become clearer (Law & Lynch, 1988). All the while, one begins to handle their binoculars skillfully and learns to attend to the right things at the right time: seeing more and differently.

The normativity of the practice lies in coordinating the person, the binoculars, and the book in the field, often with the help of others. With effort, novices can find their way among many interrelated significances. They become competent participants in the practice of bird watching. A skilled bird watcher, like a farmer or a cook or a mathematician, can thus experience the world as differentiated through their practice *directly*. Becoming an expert, to repeat, does not mean that one has extracted all the “content” of the book and “internalized” it. There is no rule for conduct or information in the pages traveling between mind and world. Instead, the participant is letting their (perceptual) activity be organized by the practice that involves them further with the world, thus achieving and maintaining communal norms of attention.

To summarize, practice theory and ecological psychology together allow us to think that values, affects, and norms are not representational states of content inside each individual's head. Instead, they are directly experienced features of the shared world, embodied by the ways people act; what they say, ignore, or notice. We continuously experience a meaningful world, filled with possibilities for action, tied to things in places and practices. The next question thus is: how do we come to experience such a world and ourselves with it? George Herbert Mead's work allows us to think that, with the right skills, we can learn to notice our shared material practices responding to us.

## 4 Hearing one's self in terms of the other

To see how we learn to notice a *self*, we now finally turn to Dewey's friend and colleague George Herbert Mead. Mead's work fits well with the temporal relationalism we brought out in Dewey's work in Section 1, and his work is highly influential in sociology for his theory of self‐formation (e.g., Abbott, 2020; Archer, 2003; Blumer, 1969; Crossley, 2011; Habermas, 1992; Noble, 1981; Nungesser, 2020; Shotter, 2017; Zahavi & Zelinsky, 2023). Yet, Mead is not engaged with in ecological psychology beyond an occasional mention (see, e.g., Costall & Still, 1989; Costall, 2004; Heft, 2007). This is a strange blind spot, as the field grew out of pragmatist thinking in which Mead played a formative role (Heft, 2001). A reason might lie in Mead's work being easily read in a representational way. With the advent of cognitivism, this became the dominant interpretation of many precognitivist works. Lev Vygotsky's theory of internalization notably suffered a similar fate (e.g., Vygotsky, 1962; 1978). Vygotsky's is seeing a reappraisal in light of recent advances in embedded and embodied cognition (Colelli, Di Bernardo, & Verde, 2025; Di Paolo et al., 2018; see also Baggs, 2015; Still & Costall, 1991, see Concluding Remarks). Here, I offer a relational reading of Mead to bring his work into the fold. I do so because Mead's theory is particularly well‐suited to understand in temporally reciprocal terms, and because the vocabulary Mead introduces can be put to use to bridge the ecological and practice‐theoretical concepts that I introduced above.

Mead resists assuming an irreducible subject behind the curtain, out of which our experience of the world is built up. Other animals will have a “bare thereness of the world” (Mead, 1934, p. 135). In ecological terms, they experience a meaningful world of affordances. As situated creatures, we humans too can feel pain or pleasure, experience an emotional episode, or remember the past “without [these] being an experience of the self” (ibid.). We can have a great variety of experiences without assuming there *must* be an intrinsic self behind those experiences to make these experiences possible (i.e., a form of reification, see Section 1). Differentiating such experience into *someone* experiencing on the one hand and *something* experienced on the other hand is itself an additional experience that may only be available as a contrast after the initial “bare thereness” of pain or of a vivid memory, and only given a history of engaging with particular norms of attention within particular practices. Under what conditions, then, do we experience the self? First, we need to draw attention to the selective (Section 4.1) and fortifying (Section 4.2) role of languaging in becoming a self. We will then consider the role of practices in more detail (Section 5).

### 4.1 Selective continuations

Languaging activities play a crucial role in differentiating the world as we go. This is because its selectivity affords us to coordinate some of the largest and smallest timescales of activity with each highly transient utterance. As for the first, we saw in the previous section that languaging is a part of our practices, of shared ways of doing things with things in places and settings. Utterances are continuations of a larger‐scale organization. That is, for languaging to shape a shared situation, all those who hear a person's utterances agree, in practice, on what it affords. Both participants are already sharing in a practice before they enter into a conversation or behavior setting (as depicted in orange in Fig. 3).

At the smallest scale of involvement (i.e., the blue lines in Fig. 1), there is also something curious about languaging. Our utterances are equally present for all in ears range. That is, both the listener and the speaker of the utterance hear the same voice (this holds equally for the use of sign language; see Mead, 1934, p. 67, 1912, p. 405, 1925, p. 271). This is not the case for the pain of a burning candle, for example. A teenager playing with a flame during dinner and getting burned has a very different experience from their mother annoyed at witnessing the same event. But when someone is talking, their ear “reveals to him his own vocal gesture in the same form that it assumes to his neighbor” (Mead, 1912, p. 403). When the mother turns to her teenage son and says: “stop playing with the candle, and act your age!” she hears her own utterance roughly as her son does. Whether the speaker or the listener, that is, utterances and gestures are equally available as they are added to the shared situation.

Taken together, however, these two features of languaging lead to a puzzle. If the meaning of an utterance is intimately connected to the practice that participants already share, and hence is broadly the same for all participants, and if languaging, moreover, unlike a painful finger, is available equally to all, whether speaker or listener, then why do people respond very differently to what was said?

Indeed, why does no one else cower in pain as a small child starts crying after burning their finger? We can readily make sense of the different responses of different people to the same utterance or gesture by acknowledging each person's history of prior involvement, to which the current situation is the newest addition (see, e.g., Fig. 2). Their histories are increasingly idiosyncratic, yet along them, much of the world is shared with others in reciprocal dependencies across situations, behavior settings, and practices. While a grocer, for example, has a different role in the store than a customer has, they share in the behavior setting and both do what is appropriate to keep the grocery store going. Likewise, politicians once were able to “agree to disagree” in debate, as they thus maintain their political practices together.

It is in a context of having *distinct* histories within *shared* worldly organizations that meaningful situations, conversations, behavior settings, and so on are made time and again together. Languaging does not add determinate meanings, but codetermines the situation by selectively continuing it. That is, their added utterances “affect the organism as they affect other organisms” (Mead, 1934, p. 145, see 1912, p. 403), because these utterances redirect the situation that constitutes both participants’ further doing and noticing.

But what the situation thus comes to afford each participant is not yet fully given. It depends in part on the different histories that were jointly redirected. Indeed, it is because the mother and the child share in a way of doing things which includes expressing pain, consoling, and so on, that the child cries out and the mother responds appropriately. That the listener of an utterance typically does *not* respond in the same way to that utterance as the speaker does, despite *in practice* agreeing on its meaning, is an engine of distinguishing various others and, ultimately, differentiating those others from a self.

We engage in some behavior settings only with certain people. Children mostly see the same teacher in class day in and day out. At work, one meets colleagues, but never one's mother. We learn basketball with our teammates. On the street, we meet strangers more than acquaintances. At a party, we talk with acquaintances more than with strangers, and so on. Teachers, friends, siblings, or parents: a child grows up with others as they continue their shared world of practices together. Everyone will surely have a unique developmental trajectory, with different people close to them at different points in time in their lives. But all of us disproportionately engage some specific people in specific situations, settings, and practices, and not others. These “significant” others typically include caregivers, siblings, and friends, with whom we share more of our histories. Across situations and specific settings, in short, we differentiate others and make kin.

There is one all‐important consequence of this differentiation of others across practices and settings that I have not yet mentioned. Recall that our utterances were equally available to anyone within ears range—whether listener or speaker. This means that while across a lifetime one may hear some people more than others, there is one voice to which all the other voices respond. Across the multitude of others we engage with, in an endless number of conversations and settings, one hears *oneself* invariably across all those involvements (Mead, 1912, p. 403). Or rather, to be precise, we learn to differentiate that invariant voice *as our own* in the mutual involvement with others.

### 4.2 A self‐selecting discussion

How do we learn to differentiate the invariant voice that accompanies our situated actions as our own? Consider a child familiar with the behavior settings of classrooms and their own teacher. This history of involvement enables the child to play at being a “teacher,” having their friends play other “pupils.” A child might tell the other children to be quiet, to raise their hands, or sit up straight, mimicking the tone of voice of their own teacher as they do. Such activities create a new shared situation, a situation of play, which selectively continues aspects of the classrooms that all children are familiar with. In that context, the activities of the “pupils” help determine a (playful) situation that determines them: inviting different responses from the children playing “pupil” from the one playing “teacher.” In Mead's words: “The response which [the child] has a tendency to make to these stimuli organizes them” (Mead, 1934, p. 150). That is, when playing together, the children's mutual adjustments to each other constituted a new organization, a situation of playing “teacher and pupil,” which sets up attentive norms to which each player needs to adapt. They thus respond in accordance with their role emerging in that situation.

Playing “teacher and pupil,” the children set up a joint situation that sets them all up to act as pupils or as teachers. They, in effect, explore the various roles of that behavior setting. Those activities of responding in the role of another reciprocally continue their developmental trajectory. Crucially, however, a child can also learn to set up such a situation all on their own. Indeed, children often play alone: a child may then switch roles continuously to first be an indolent pupil and then a corrective teacher:

The child says something in one character and responds in another character, and then his responding in another character is a stimulus to himself in the first character, and so the conversation goes on (Mead, 1934, p. 151).

**Fig. 4:**
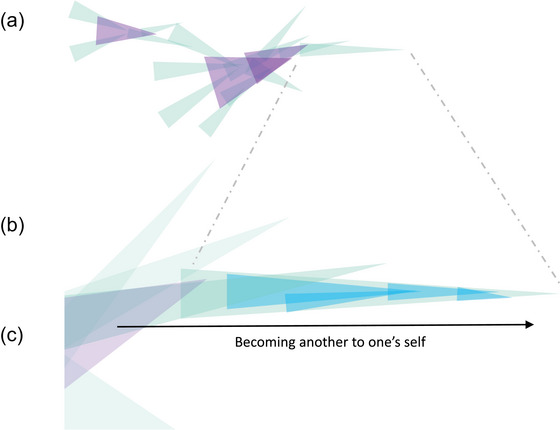
Bundling activities into practices over time. (a) A conversation followed by a joint situation of playing “teacher and pupil” direct one of the children. This picture draws on Fig. 3A, suppressing the overarching teaching practice for the moment. (b) Zooming in on one of the children as it starts to play the same game alone, by talking to themselves as first pupil, then teacher, and so on (the small blue lines). Notice the organizational similarity with Fig. 1c: the child sets up the conditions for their own continuation, while giving shape to a larger‐scale coordination (the larger green line). Just as a child within this process can directly experience the potential burn, so too can a child learn to anticipate their own voice's response. The larger timescale is constituted by these responses, and each new utterance adds to and maintains it (see text for details).

Given enough experience in classrooms or with friends, a child might set up such a situation on their own. Hearing themselves say things belonging to one position in a behavior setting (i.e., the teacher), their utterances form the conditions to respond to in the place of another's position in the behavior setting, and so on. They lay down a path in talking all by themselves. With enough experience in the shared world of behavior settings, they learn to anticipate the response, the doing and saying, of another, and subsequently take those roles on too. Over time, the child thus takes over different positions in a conversation—overtly, by being speaker and listener at once—and responds to themselves in terms of the other (see Fig. 4).

A child hearing and talking to themselves is *selectively continuing* an aspect of their developmental coordination by responding to their “own” utterances, instead of, say, a toy or a phone in front of them, or their father calling. The conversation that unfolds as the child switches roles over time sets up the conditions for its own continuation: attending to and propagating the voice that invariantly accompanies all their involvement with others, across behavior settings and practices. Talking out loud to herself, the child sets up the conditions for a developmental timescale that organizes her subsequent utterances and gestures in a particular way.

Accordingly, Mead writes, a “certain organized structure arises in him and in his other which replies to it, and these carry on the conversation of gestures between themselves” (Mead, 1934, p. 151). As I have tried to stress, this “certain organized structure” is unfolding out in the open. It is the larger temporal scale of coordination that makes up part of our reciprocal relation with the world—a process continuously getting ahead of itself (see Fig. 4b). And just as we, over time, learn to experience the potential futures of that worldly process directly, experiencing a flame as “light‐that‐means‐pain‐when‐contact‐occurs,” so too can we experience the future of a conversation with ourselves: inside this coordination, we anticipate how our voice might respond to what it is saying now *as* it is saying it now.

Talking to oneself over time, to Mead, “is the simplest form of being another to one's self” (Mead, 1934, p. 151). That is, the *self* that arises in this example is the extensive worldly organization available to the child as her coordination with her environment prolongs and refines: by selectively noticing and stimulating this invariant voice to respond, we set up what might be called a “self‐selecting” process. Thus, this voice progressively forms the conditions for subsequent experience. As we respond to our own voice, we selectively continue and increasingly fortify a self. Such a self is a temporalized phenomenon, involving past, present, and future at once. In our selective involvement with our past, we hear ourselves talk and, codetermining a larger timescale, experience directly what we are going to say next. We anticipate, that is, our *own* future response.

## 5 Supersizing others, supersizing the self

It is not just individual others, teachers, fellow pupils, and so on that respond to a person's doings and sayings. Across all our worldly involvements, the directional tendency of a setting or even a practice becomes available as the person's skills at navigating them grow, and they respond to one's unique additions to them. Consider gaining a sense for the normative requirements of the practice of basketball. To learn what one ought to do, a child needs to engage with their teammates in concrete behavior settings. Each child getting their attention educated by the others, thus a child learns that he:

can throw the ball to some other member because of the demand made upon him from other members of the team. … He has their attitudes, knows what they want and what the consequence of any act of his will be, and he has assumed responsibility for the situation (Mead, 1934, p. 175).

The player “has their attitudes,” again, because his actions shape the larger setting that shapes all of the players, so that they experience the setting's (joined) possibilities directly (recall Fig. 2). Playing basketball, our attention has been educated to notice teammates opening up, say (e.g., Araújo, Davids, & Hristovski, 2006). Passing the ball successfully is encouraging, and feels good, in part because it continues the game. Indeed, during a basketball game, failure to do or notice the appropriate thing results in, say, losing the ball and in scorn from their teammates, frowns, and utterances of dismay (“I was wide open!”) from their friends. Participating in the wider process, the child senses the affective tones and the normative requirements of their shared situation. That is, they experience what a basketball game affords directly in the voices and actions of those close to them.

Above, I stressed that practices do not depend on any particular person. Instead, practices, and the behavior settings within them, have a distinct (temporally reciprocal) organization constituted by many different participants doing and saying things with things. That overall organization, in turn, organizes what its participants do, say, and notice. In Mead's words:

The attitudes of the other [participants] which the participant assumes organize into a sort of unit, and it is that organization which controls the response of the individual. … We get then an “other” which is an organization of the attitudes of those involved in the same process (Mead, 1934, p. 154).

To translate this into our vocabulary: the activities of doing, saying, and noticing organize into “a sort of unit,” which we have called a *practice*. That practice embodies norms of attention, which are constituted by the activities of a multitude of individuals, yet is reducible to no one in particular (see Section 3.1). As an individual learns to engage with these practices, they become responsive to some possibilities rather than others (cuing up at the pharmacy, never thinking of dancing on the counter), made to notice certain things while ignoring others (seeking eye contact to determine who is last in line, failing to notice how danceable the song on the radio is). That is, the practice starts to shape (or “control”) the response of the individual.

Crucially, Mead emphasizes that these organizations are made by the ways of doing things of multiple “other” people. Together, these temporally reciprocal processes then gain an overall tendency which directs their participants to notice the right things at the right times (much like we saw in the case of bird watching above). This overall tendency is what Mead called the *generalized other*: “The attitude of the generalized other is the attitude of the whole community” (ibid.).

Although it might again be tempting to read this “organization of attitudes” as an inner organization, with Schatzki and Reckwitz but also Gibson and some of his followers (e.g., Costall, 1995; Crippen, 2022; Heft, 2001; Noble, 1981), we can understand these meaningful organizations as being already available in the environment in which we participate. Indeed, in passing, James Gibson himself acknowledges the generalized other as an “ecological object” (J. Gibson, 1979, p. 135). That is, behavior settings, but also practices, become available for direct experience as *generalized others* that respond to our involvement with them in particular ways over time. To see how this works, let us turn to an example.

### 5.1 Hearing our practices speak

Consider the Dutch educational system. Children as early as 5 years of age are assessed and graded, rewarded, and compared to their peers every day. They meet with their parents’ and teachers’ (dis)approval in different ways—as do parents among each other. At 11−13 years of age, children get tested and sorted into “higher” or “lower” forms of education, affecting the rest of their lives. If these children are prepped to go to university, the competition has only just begun. Beyond the classroom, for instance, commercial providers promise to improve school performance, and “good” parents sign their children up for such extracurricular classes if they want them to succeed (and are able to afford it). This highly organized “way of doing things” is what children (and parents) need to grow sensitive to if they are to thrive in school. How do these norms of attention reorganize our experience?

If all is well, and we follow the overall tendency of the practices, we might not experience much of these practices beyond an immediate sense of appropriate action. We are then working along with the tendency of the “generalized other”—opening up ever new possibilities for action as we go. But as things become difficult, if it is hard to maintain coordination with a practice over time, the affective atmosphere of a practice may grow salient, and we find our*selves* as standing over and against the generalized *other*.

The generalized tendencies of our practices form the conditions for developing as a person, shaping what they do and notice, and what one finds significant. In this case, the self takes shape along the large‐scale generalized tendency of the educational system. The generalized other directs a child's current involvement (i.e., it educates attention). But, importantly, it does so by providing much more long‐term norms of attention along which they are becoming a self (Fig. 4). That is, the practice in which they grow up is increasingly differentiated as responding to some*one* that the child is learning to selectively continue: Mead called this someone the “me”:

there arises an organized group of responses. And it is due to the individual's ability to take the attitudes of these others in so far as they can be organized that he gets self‐consciousness. The taking of all of those organized sets of attitudes gives him his “me”; that is the self he is aware of (Mead, 1934, p. 175).

In our example, the organized attitudes are the practices of an overly meritocratic educational system as it responds across settings and places, in the voices of teachers, parents, and friends, to *one* person's added activities. That is, the generalized other shapes the acting person's developmental trajectory into a unique organization: a *me*. The *me* progressively takes shape as the self‐selecting child, all the while, is set up to see their own activities in light of their shared practices. In this case, in light of the exceedingly high demands and expectations of everyone, yet no one.

Dutch youth reports experiencing the pressures of the educational climate; feeling they need to be perfect, feeling burdened by responsibility for their own success, and afraid to disappoint others (Schoemaker et al., 2019; see De Looze et al., 2020). These feelings are not merely in their heads. Rather, these youths express a direct experience of the practice in which they participate—and how it responds to *their* involvement. It can be likened to the experience of sensing a conversation getting away from you and slipping into a fight; while having no control of the conversation, one is able to sense the tension because one is taken up in it. Participating in a practice likewise does not mean one is able to control much of it. Still, it allows anticipating the response of the overall practice to one's continued involvement. That response shapes the self. As they grow up in a competitive climate, a child may feel the expectation of everyone, yet no one in particular: shaping their sense of self‐worth in terms of and relative to the generalized other in which their *me* develops.

As a *me* develops in increasing involvement with others, the self‐selecting process considered in Section 4.2 continues. Indeed, these two reciprocal processes run together. The next‐to‐final section will bring the previous ideas together and consider how the worldly involvement with *others* is making a *me*, while this *me* is making a *self*.

## 6 The self as an *I*‐*me* reciprocity

Temporalizing worldly experience was the principled step to get us out of our heads: rather than thinking that our worldly involvement was the effect of inner states of content about the world, we could think of the temporal reciprocity, this indeterminate yet forwardly directed relation, as experienced directly. The claim is that the self forms in a reciprocal structure with the shared world of practices, with its various others, all responding to someone. I have stressed that temporally reciprocal processes get ahead of themselves, continuously invited to act by their environment, expanding into new possibilities as they go. Do we have anything in Mead's work to match this unfinished, future‐directed tendency? Indeed, we do. In Mead's technical vocabulary, the future involvement is performed by an indeterminate *I* getting ahead of one's actions.

The *me* is of a larger timescale and sets up the conditions for the *I* to continue involvement. Conversely, engaging many shared settings and situations over time with (significant) others, the *I*’s responses form a *me* over developmental time. This reciprocal process lays down a unique history of engaging the shared world, shaping attention as the relation gets ahead of itself and requires further activity to determine it anew. This determining act is performed by the *I*, which is set up by and continues a *me* that itself continues to take shape in a reciprocal relation with various (generalized) others:

[It] is the presence of those organized sets of attitudes that constitutes that “me” to which he as an “I” is responding. But what that response will be he does not know and nobody else knows. … The response to that situation as it appears in his immediate experience is uncertain, and it is that which constitutes the “I” (Mead, 1934, p. 175).

Mead's temporal structure of the self can be read as a refinement of the developmental trajectory that forms a person's life as it grows up in the reorganized world that this paper foregrounded. That is, the *I* is only available upon reflection, as it is taken up in the *me* by an *I* already slightly ahead of it again:

If you ask, then, where directly in your own experience the “I” comes in, the answer is that it comes in as a historical figure. It is what you were a second ago that is the “I” of the “me.” It is another “me” that has to take that rôle (Mead, 1934, p. 174).

**Fig. 5:**
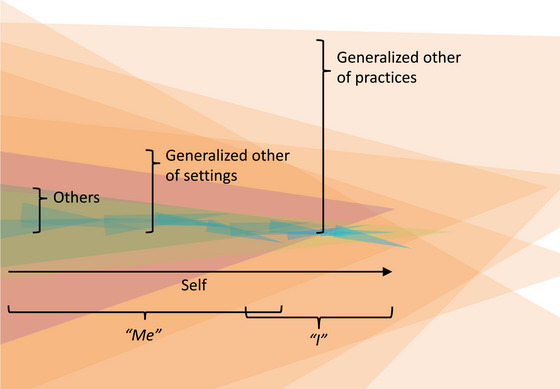
Zooming all the way in on an acting person within the reorganized world, all the timescales that we considered in the previous section come in play. The smallest timescales are indicated in blue: the situated activities of an individual. The largest timescales drawn are the practices in which the individual has learned to participate (in orange). These have given rise to a behavior setting, say a casual dinner, indicated in purple. All of these are shaping the developmental coordination of the person (in green), setting up the conditions for further activity: the blue lines all the way to the right. In an enlanguaged world, those trailing off blue lines are the “I” which is set up by the practices, settings, conversation, and so on that form generalized others vis‐à‐vis the invariant sayings and doings of a “me.” The me is the ongoing experience of engaging shared activities with “others,” and continuously sets up the conditions for the acting “I.” The I, meanwhile, selectively continues the me in relation to others with whom it shares the world.

Mead's theory of individual development can be fruitfully read as “enlanguaging” the temporally reciprocal relation that Dewey (and Gibson) highlighted. The temporally reciprocal relation with an enlanguaged world of practices and settings, shared with many (significant) others, constitutes an individual's life over developmental time, and transforms worldly experience to that of a *me* and its *I* getting ahead of it. In Mead's words, “the ‘I’ both calls out the ‘me’ and responds to it” (Mead, 1934, p. 178). See Fig. 5 for a schematic summary of the relations among the various concepts introduced.

The *I* and *me* are, in short, temporally reciprocal. To be precise: (a) the larger scale world‐involving coordination with others forms a *me* which sets up the conditions for an acting *I*, while (b) the *I* achieves continuation of that *me* in a particular way, which adds to the larger worldly coordination with one's (significant) others. Notice, then, that while the *I* is reciprocally dependent on a *me*, the *me* is, in turn, reciprocally dependent on sharing the world with many others across situations. There is, in short, no sense in which the *I* is an inner or prior subject. The *self* is a temporally reciprocal process, an “I‐me reciprocity,” which forms out of and contributes to the shared world of all others.

Think of a teenager's stream of experience while seated in class. She needs to pay attention to the teacher explaining a math problem. The teacher might prompt her to explain at any moment. Indeed, she feels anxious. Her grades are dwindling, and she is growing unsure if she is able to pass this year. She has seen her parents worry, even though they try to be supportive. Her younger brother does much better and teases her at dinner. She is not looking forward to that. Meanwhile, she finds her mind has wandered again—staring out the window of the classroom, where she is the only one suddenly noticing a Mistle thrush singing. She will get to go out again birding with her father this weekend. “Class should be over soon,” she might think, “Isn't the bell about the sound? I hope the teacher passes me over this time.”

If you followed the analysis this far, you might recognize that nothing in this everyday scene requires the teenager to revert to an inner realm of thought. Inside the intricate multiscale developmental coordination with the world that we considered, instead, the teenager's experience can be thought of as a tuning in and out of some of the many ongoing timescales of involvement to which she is adding her unique individual trajectory. Her *me* is shaping with the generalized others and significant others she has gotten to know: the waxing and waning of a classroom setting, the demands the teacher makes on students in general and on her in particular. These are available inside the practice's general tendency: the schooling system in response to her persistent failing to keep up. The teenager directly experiences this tendency not just as a general mood. She can also all but hear the disappointing tone of her mother's voice, or her mocking brother, both maintained across her involvement with the practice, as her mind wanders nonetheless. Insufficiently set up to follow those voices back to the classroom, her *I* gets away from her (from her *me*), and she is taken up, just for a second, in the norms of attention of birding that she is also growing up in. Her heart skips a beat as she sees the thrush on the school lawn, not noticed by anyone else.

## 7 Concluding remarks

This article argued that becoming a “self” is not about internalizing either the external world or our own actions in it. We continuously experience a meaningful world, filled with possibilities for action, tied to things in places, settings, and practices we share with others. It is not far off to say that as we develop our skills of doing and noticing what the world requires of us, we can experience the organized world of practices speaking to us. Our “self” lies in the reciprocal relation between this reorganized world and the situated activities a person contributes to it. Indeed, if recent arguments in embodied cognitive science hold up, there is nothing to internalize. What we call “internalization” is a reification of the growing and expanding relations with our shared world that this paper aimed to foreground. On the view developed here, the self cannot be understood outside of the world of practices that it forms out of and to which it contributes.

Drawing on ecological thinking, practice theory, and Meadian considerations, I argued that growing up in a reorganized world of practices, our lives are increasingly organized into *me*’s as (generalized) others are discerned for their response to us and our *I*’s response to them. In turn, each *me*, setting up the conditions for an *I* ahead of it, engages the world and others so as to organize our practices anew. This is the relational account of self‐formation. We do not each internalize the world, but our shared world individualizes us in action. The relational self is constituted in a temporally reciprocal relation with the world (e.g., Fig. 5). Such a relation does not end when we close our eyes.

With an eye for the world at large that this paper aimed to supply, it seems that reverting to some inner state of consciousness becomes superfluous. But, of course, drawing attention to the relations that make us is just the beginning. A relational instead of representational approach to individualization needs to be substantiated by empirical study. This is, in fact, a main motivation for this paper: it was not to explain individualization without internalization, but to supply a way of looking that would allow to go beyond the limits that traditional thinking imposes on our methods. Once we step away from the assumption that experience *must* be located inside the head, we can start rethinking how, where, and when to look to find the experiences that we are interested in.

If individualization is an expansion of worldly involvement, then the more personal an experience becomes, the larger the timescales of actual involvement that we also need to consider to keep the experience in view. There are various methods to gain sight of these larger‐scale processes. Whichever methods we adopt, they should enable researchers to get involved for longer and see the coordination across timescales that otherwise escapes notice (and is thus attributed to the brain instead). For example, we could try to pair an eye for an individual's responsivity to the affordances that surround them (see Section 2) with a grasp of the behavior settings where they spend much of their lives (Section 3.1; for an introduction to this method, see Lucas, 2024).

Across behavior settings, we can also look for the practices that such settings achieve and maintain. An ethnographical approach might help gain sight of the extensive involvement with materials in practice. For instance, in an effort to understand imagination without recourse to mental representation, Van Dijk and Rietveld (2020) closely observed architects in their practice. By foregrounding how architects’ skilled activities codetermine multiple timescales of shared involvement concurrently, we were able to hypothesize that imagination is not a “detached” inner process about the world, but might be approached as a direct experience of the reciprocal relationship in which multiple scales of involvement codetermined each other (Van Dijk & Rietveld, 2020; see also Trasmundi & Cowley, 2020). Such work of situating involvement across wider patterns of engagement follows a long tradition of looking for *cognition in the wild* (Hutchins, 1995).

Ethnographical findings can be integrated with data from other methods to thicken our account of an individual's experience. Cognitive archeology allows us to trace how the mind is shaped by things across even larger timescales of engagement (Malafouris, 2013). Cognitive Event Analysis, which was developed to get the coordinative function of languaging to the fore (Section 3), might serve to integrate various such data with other situated activity across timescales (Pedersen, 2012; Steffensen, 2013; Steffensen, Thibault, & Cowley, 2010). For instance, there seems a great opportunity to thus pair an eye for the world at large with observations of the real‐world context of self‐directed talk (e.g., Brinthaupt, Hein, & Kramer, 2009). Elsewhere, formal models of the dynamics of multi‐scaled systems might enable predictions of phenomena of *self* as situated and temporally extensive (Hasselman, 2023; Van Geert & de Ruiter, 2022).

While experience need neither be thought to transport into nor be contained by the brain, our nervous system is likely a *sine qua non* for most of the experiences discussed in this paper. In light of the larger‐scale dynamics in which we experience, we might also reapproach questions about the role of the physiological processes that take place inside our heads (e.g., Keijzer, Duijn, & Lyon, 2013; De Wit, 2024; Favela, 2023; Raja, 2024, for research along these lines). An adapting nervous system seems conducive to the rich forms of worldly coordination that make experience. Distributing the self across an open‐ended reciprocal relation between the shared world and self‐selecting involvement suggests an endless many different trajectories and, indeed, various ways this process might develop in ways that hinders the person involved (De Haan, Rietveld, Stokhof, & Denys, 2013; Dings & de Haan, 2022; Flanagan, 2018; García, 2023). This paper suggests that to make sense of the neural contributions to such situated phenomena requires us to expand the context in which we view them far and wide.

In so doing, the paper joins emerging efforts, notably in phenomenological‐enactive theories of mind, to avoid blinding ourselves to worldly complexity in favor of a study of inner mental states (Colombetti, 2013; De Haan, 2020; De Ruiter & Thomaes, 2023; Di Paolo et al., 2018; Gallagher, 2021; Zahavi & Zelinsky, 2023). Of particular interest are the recent attempts to connect enactivism with Vygotsky's internalization (Di Paolo et al., 2018; Colelli et al., 2025). Complementing some of the work here, Colelli et al. (2025), for instance, argued to think of internalization, not as a transfer of external structure, but as a reorganization of a person's “space of mind” through embodied and social interactions (ibid., p. 11). While they retain the proverbial “inner” to mark off the result of the process of individualizing in practices, their point is well‐taken. Although I have limited the scope of this paper to an integration of ecological, pragmatist, and practice‐theoretical considerations through Mead's work, I hope this paper may also present a new opportunity in a continued effort to bring enactive considerations and ecological thinking closer together.

Whatever the theories and methods we adopt, understanding individualization from a relational perspective requires pairing an eye for the world at large with scrutiny of concrete details of material involvement. As we saw Mead observing above, we typically do not experience ourselves as another to one's self all the time. In what situations do we actually talk to ourselves? What role do specialized perception‐words, or everyday words like “you” or “I,” play in the process of self‐selection? What practices or skills do we need to cultivate to afford some control of what we notice or to sustain a chain of thought? How might we set up the environment to avoid self‐destructive behavior? These are questions about the ecology of self that open up once we go relational. They can be answered only by getting out of our heads, getting out of the lab, and into the wider world to study the multiple conditions that make us.

## Notes

1 This discussion and Section 2.1 build on an analysis detailed in Van Dijk (2021).

2 These figures are a development of the figures first presented in Van Dijk and Rietveld (2018). They were, in turn, inspired by Ingold (2011, p. 69), and the works of Shotter (1983) and Schatzki (2002). I mean these figures to help visualize the relations between the various concepts discussed—notably, their temporal extent, their multiplicity, and open‐ended, forward‐directed character. The figures are, however, not meant to stand on their own, they are best read along with the text. It is my hope that they may, in turn, support the argument in a fruitful way (see Smith, Van der Zwan, Van Dijk, Bruineberg, & Hummels, 2023).0
